# Physicochemical Properties and Structure Changes of Food Products during Processing

**DOI:** 10.3390/foods11152365

**Published:** 2022-08-07

**Authors:** Marta Igual, Javier Martínez-Monzó

**Affiliations:** Food Investigation and Innovation Group, Food Technology Department, Universitat Politècnica de València, Camino de Vera s/n, 46022 Valencia, Spain

This Special Issue is dedicated toward the understanding of the physicochemical properties and structure changes of food products during processing. Processing food is necessary to not only extend product shelf life but also lead to changes in the physicochemical properties and structure of foods, which can be desirable or undesirable. Processing operations include dehydration, thermal treatment, encapsulation, and extrusion. The physicochemical properties of food are mainly responsible for the final quality of the product. Moreover, the measurement of these properties is important for design and quality control during the processing of the food.

The physicochemical and structural changes in food during processing depend on the type of food being processed (solid or fluid) or its constituents. There are numerous physicochemical properties of food, for example, hydration properties (water activity, water absorption capacity, water retention capacity, hygroscopicity, dispersibility, solubility, etc.), rheological fluid behavior, mechanical properties, optical properties (color, translucence, etc.), and thermal properties. However, each type of food needs to be characterized by studying certain specific physicochemical properties. The choice of the most appropriate physicochemical properties in each case is very important in food research.

Physical and chemical changes in each constituent and ingredient result from processing operations and often lead to physical, sensory, and nutritional changes in food, and therefore, in the quality.

In the present article collection, there are 14 articles of high scientific value. Several of them have evaluated the extrusion process to obtain new snacks or ingredients [1,2,3]; fermentation has been studied in others as a food process [4,5]. The physicochemical and structural properties of food can be affected by the addition of ingredients, such as those studied in a different article of the present Special Issue, for example, the impact of resistant maltodextrin addition on the physicochemical properties in orange juice [6], the effect of pea protein and insects addition on the physicochemical properties and consumer acceptance of bread [7], and the use of milk fat/cellulose ether emulsions in spreadable creams and the effect of in vitro digestion on texture and fat digestibility [8]. The effect of changes in the physicochemical and structural properties of foods in sensory terms has been evaluated by Campus et al. [9], who studied the influence of almond variety on color, chemical, physical, and sensory characteristics of “amaretti” cookies during their shelf life. Authors showed how the choice of the variety to be used in this type of product is extremely important, directing producers and processors toward those with the best aptitude for transformation, given the same agronomic performances.

Gluten-free products available on the market have a low textural quality associated with the high crumbly structure, low flavor, aroma, poor mouthfeel, lesser appearance in comparison with the conventional final baked products. The aim of Chis et al.’s work [4] was to assess the influence of rice sourdough fermented with the *Lactobacillus spicheri* DSM 15429 strain on textural, volatile profile, and sensorial properties of gluten-free muffins in order to obtain baked goods with improved quality characteristics. Lactobacillus spicheri is a novel strain isolated from industrial rice sourdough but unexploited for bakery products manufacturing. The results showed that *Lactobacillus spicheri* DSM 15429 was able to grow in the rice flour, influencing the texture and the volatile profile of gluten-free muffins as well as their sensory characteristics. Both textural parameters and volatiles recorded significant differences compared to muffins obtained with a spontaneously fermented rice sourdough. The hardness and cohesiveness decreased, while the springiness and resilience of gluten-free muffins improved their values. The volatile profile of gluten-free muffins was significantly improved by utilization of the rice sourdough fermented with *Lactobacilus spicheri* DSM 15429. 3-methylbutanal, 2-methylbutanal, acetophenone, and limonene were the main volatile derivatives responsible for the aroma and odor scores of the sensory analysis.

The interest in plant-based products is growing in Western countries, mostly due to health and environmental issues that arise from the consumption and production of animal-based food products. Many vegan products today are made from soy, but the drawbacks include the challenges of cultivating soy in colder climates, such as northern Europe. Therefore, the study of Zahari et al. [3] investigated whether industrial hemp (Cannabis sativa) could substitute soy in the production of high-moisture meat analogs (HMMA). A twin-screw co-rotating extruder was used to investigate to what extent hemp protein concentrate (HPC) could replace soy protein isolate (SPI) in HMMAs. The substitution levels of HPC were 20 wt%, 40 wt%, and 60 wt%. The pasting properties and melting temperature of the protein powders were characterized by the Rapid Visco Analyzer and Differential Scanning Calorimeter, respectively, and the produced HMMA was analyzed by determining the texture and color attributes. The results showed that it is possible to extrude a mixture with up to 60% HPC. HPC absorbed less water and needed a higher denaturing temperature compared to SPI. Increasing the moisture content by 5% would have resulted in a reduction in hardness and chewiness. The lightness (L* value) was found to be significantly higher in the SPI product and decreased in the mixture with higher HPC (*p* < 0.05). Additionally, Kaleda et al. [5] indicated that plant materials as meat analogs are often nutritionally incomplete and also contain antinutrients, thus necessitating the exploration of alternative plant proteins and pre-treatments, and demonstrated the application of phytase and fermentation to a pea-oat protein blend with a good essential amino acid profile and subsequent texturization using extrusion cooking. Along the same path, García-Segovia et al. [7] studied the incorporation of plant- and insect-based protein sources in wheat-based formulations. From this work, authors indicated that including pea protein or insect powder improved the nutritional value, increasing protein content, but influenced the dough and bread properties. Pea protein significantly increased the dough extensibility, tenacity, and their ratio in dough with insect blends and the control. Bread texture properties were significantly affected by the addition of pea and insect flour. Higher amounts of pea protein incorporation increased the hardness values and showed a mean cell area lower than the control bread. Crust color analysis showed significant differences concerning the control bread, while crumb color was affected by the flour color. Word association analysis showed insect bread was associated with an emotional dimension, wheat bread was linked with “tradition”, and pea bread was associated with “fruit and vegetable”.

Espert et al. [8] investigated the texture properties and fat digestibility of new spreadable chocolate creams formulated with an emulsion composed of milk fat and a cellulose ether as a fat source. The spreadability was analyzed at 20 °C and compared with a commercial spreadable cream formulated with palm fat. Structural changes in the creams after the in vitro oral and gastric digestion stages were evaluated; lipid digestibility was determined by titration with NaOH during intestinal digestion. Spreadability tests showed the spreads were similar. After oral digestion, the commercial spread showed an increase in extrusion force because of flocculation induced by saliva, an effect not observed in spreads with cellulose ether. Digestibility determination showed lower values for the reformulated spreads. Therefore, milk fat-cellulose ether-based emulsions offer an alternative to achieve reformulated spreadable creams, with physical properties similar to those of commercial products but providing reduced fat content and lower lipid digestibility, without compromising the quality of the final product.

The physico-chemical and microstructural changes of “Rojo Brillante” persimmons in two maturity stages were evaluated during air drying in Vilhena et al.’s article [10]. Authors indicate that the maturity stage influences fruit characteristics during the drying process. The formation of a secondary epidermis during the drying process, concomitant with internal flesh gelification, was related to the moisture loss that occurred in the fruit in each maturity stage. Finally, this study revealed that “Rojo Brillante” persimmon is a suitable astringent variety to be submitted to a drying process after taking into account that the final product characteristics depend on the maturity state upon harvest.

The role of roasting in cold brew coffee chemistry is poorly understood. The brewing temperature influences the extraction processes and may have varying effects across the roast spectrum. To understand the relationship between brew temperature and roast temperature, the group of Rao prepared hot and cold brew coffees from Arabica Colombian coffee beans roasted to light, medium, and dark levels [11].

The study of O’Donoghue et al. [12] studied the α-relaxation temperatures, derived from the storage and loss moduli using dynamic mechanical analysis, which were compared to methods for stickiness and glass transition determination for a selection of model whey protein concentrate powders with varying protein contents.

The Special Issue also contains interesting works, such as the article published by Gaspare et al. [1], which studied the effect of tiger nut inclusion in the formulations on relevant physiochemical characteristics of the extruded snacks. With their results, authors affirmed that the incorporation of 10% tiger nut flour into rice-based formulation was suitable for making gluten-free snacks with acceptable physical properties. Oh et al. [13] studied the retrogradation properties and kinetics of rice cakes with the addition of glycerol and sucrose fatty acid ester and observed that when glycerol and sucrose fatty acid ester were added to rice cake, they interfered with the starch re-association and recrystallization. Glycerol acted as a plasticizer, interacted with water and starch molecules, improved the water-holding capacity, and inhibited the starch retrogradation in macroscopic and microscopic levels.

Schmid et al. [2] analyzed the effect of the process parameters and the extent of thermo-mechanical treatment on the structural and functional properties of apple pomace after extrusion trials using various screw speeds, water contents, and barrel temperatures.

The work of Arilla et al. [6] aimed to evaluate the addition of resistant maltodextrin on the physico-chemical properties of pasteurized orange juice (with and without pulp). This study concluded that resistant maltodextrin addition in a wide range of concentrations is feasible from a food technology viewpoint. However, the optimal dose of resistant maltodextrin will depend on the functional effect to be achieved. Differences in particle size distribution were observed due to pulp content.

Finally, the rheological properties of twelve different licorice root extracts were evaluated using a rotational viscometer as a function of soluble solids content (15–45 °Bx) and temperature (30–70 °C) by Nasiri et al. [14].

Concluding the presentation of this Special Issue, we would like to thank the abovementioned research teams for their contributions to the present article collection, which provide excellent examples of the multidisciplinary character of the research on physicochemical properties and structure changes of food products during processing.
